# Three-dimensional volumetric assessment of hard tissue alterations following horizontal guided bone regeneration using a split-thickness flap design: A case series

**DOI:** 10.1186/s12903-023-02797-3

**Published:** 2023-02-22

**Authors:** Daniel Palkovics, Eleonora Solyom, Kristof Somodi, Csaba Pinter, Peter Windisch, Ferenc Bartha, Balint Molnar

**Affiliations:** 1 Department of Periodontology, Semmelweis University, Szentkirályi Street 47, Budapest, 1088 Hungary; 2 Empresa de Base Technológica Internacional de Canarias, S.L., Alcalde Jose Ramirez Bethencourt Avenue 17 Las Palmas De Gran Canaria, 35004 Las Palmas De Gran Canaria, Spain

**Keywords:** 3D radiographic evaluation, CBCT segmentation, Subtraction analysis, Horizontal ridge augmentation, Split-thickness flap

## Abstract

**Objectives:**

To analyze morphological, volumetric, and linear hard tissue changes following horizontal ridge augmentation using a three-dimensional radiographic method.

**Methods:**

As part of a larger ongoing prospective study, 10 lower lateral surgical sites were selected for evaluation. Horizontal ridge deficiencies were treated with guided bone regeneration (GBR) using a split-thickness flap design and a resorbable collagen barrier membrane. Following the segmentation of baseline and 6-month follow-up cone-beam computed tomography scans, volumetric, linear, and morphological hard tissue changes and the efficacy of the augmentation were assessed (expressed by the volume-to-surface ratio).

**Results:**

Volumetric hard tissue gain averaged 605.32 ± 380.68 mm^3^. An average of 238.48 ± 127.82 mm^3^ hard tissue loss was also detected at the lingual aspect of the surgical area. Horizontal hard tissue gain averaged 3.00 ± 1.45 mm. Midcrestal vertical hard tissue loss averaged 1.18 ± 0.81 mm. The volume-to-surface ratio averaged 1.19 ± 0.52 mm^3^/mm^2^. The three-dimensional analysis showed slight lingual or crestal hard tissue resorption in all cases. In certain instances, the greatest extent of hard tissue gain was observed 2–3 mm apical to the initial level of the marginal crest.

**Conclusions:**

With the applied method, previously unreported aspects of hard tissue changes following horizontal GBR could be examined. Midcrestal bone resorption was demonstrated, most likely caused by increased osteoclast activity following the elevation of the periosteum. The volume-to-surface ratio expressed the efficacy of the procedure independent of the size of the surgical area.

**Supplementary Information:**

The online version contains supplementary material available at 10.1186/s12903-023-02797-3.

## Background

The occurrence of horizontal and vertical alveolar ridge reduction following tooth extraction is well-reported in the literature and supported by several animal and clinical studies [1–3]. In instances where an alveolar ridge preservation (ARP) procedure [4] is not performed, or the residual ridge dimensions following ARP do not allow implant placement, subsequent ridge augmentation must be performed to reestablish the lost alveolar ridge dimensions [5, 6]. Horizontal ridge augmentation techniques include guided bone regeneration (GBR) [7], onlay block grafting [8], and ridge splitting [9]. All these techniques are well-researched and can be used predictably to restore the width of the alveolar ridge [10].

Since the original description of the GBR concept [7], its effectiveness to reestablish the alveolar ridge width before implant placement has been proven many times. Many different types of regenerative materials and flap techniques have been described [11, 12]. However, the application of resorbable collagen membranes is becoming increasingly preferable over non-resorbable membranes due to their superior handling capabilities and simpler complication management [13]. Besides regenerative materials, the surgical procedure applied plays a crucial role in the success of the intervention. Alveolar ridge deficiencies can be accessed from either a full-thickness mucoperiosteal flap [11, 14] or a split-thickness flap [15–17].

Hard tissue alterations following horizontal GBR procedures can be validated with either clinical or radiographic methods. Due to its wide accessibility, radiographic evaluation is performed on cone-beam computed tomography (CBCT) scans taken before augmentation and after the healing period [18]. Three-dimensional (3D) models can be generated from CBCT datasets, allowing measurement and visualization of volumetric changes [19–27].

In a previous article, our research group described a semi-automatic CBCT segmentation method [28]. This method was used to assess volumetric and 3D morphological hard tissue alterations following alveolar ridge preservation [29] and regenerative periodontal surgery [30]. With this method, previously unreported aspects of postoperative hard tissue changes could be demonstrated. Consequently, to avoid undesirable morphological hard tissue changes and achieve a more favorable result, surgical procedures could be altered in the future.

Accordingly, the primary aim of the current investigation was to describe a thorough radiographic method for the 3D evaluation of hard tissue changes after staged horizontal GBR. The secondary aim was to radiographically analyze morphological, volumetric, and linear hard tissue alterations following horizontal GBR using a split-thickness flap design.

## Materials and methods

### Study design

This prospective single-center consecutive case series included a total of 10 surgical sites on the lower jaws of eight patients from an ongoing prospective randomized clinical trial investigating the efficacy of horizontal GBR before dental implant placement. The study followed the PROCESS guidelines checklist (originally published in 2016, revised in 2018) [31]. The study protocol was approved by the Semmelweis University Regional and Institutional Committee of Science and Research Ethics (Approval Number: SE RKEB 145/2018) and the U.S. National Library of Medicine (www.clinicaltrials.gov; trial registration number: NCT05538715; registration date: 09/09/2022). The study was conducted in full accordance with the Declaration of Helsinki of 1975, revised in 2013 [32]. Surgical interventions were performed with the understanding and written informed consent of every participant. All surgical procedures were carried out by expert clinicians, each with over 15 years of experience in the field.

### Patient selection

Patients with good compliance and proper oral hygiene were enrolled who had missing single or multiple premolars or molars, where horizontal ridge augmentation was necessary to provide favorable functional and esthetic outcomes for prosthetically driven implant placement. The exclusion criteria were (i) general medical conditions: previous irradiation therapy in the maxillofacial area, uncontrolled diabetes, uncontrolled high blood pressure, systemic steroid treatment, systemic bisphosphonate treatment, pregnant or lactating women; (ii) age: < 20 years, (iii) smoking; (iv) periodontal status: untreated periodontitis with high levels of residual inflammationa full mouth bleeding score (FMBS) of > 25%; and (v) oral hygiene: a full mouth plaque score (FMPS) of > 25%.

CBCT images were taken with a Planmeca ProMax 3D Plus and a Planmeca Viso G7 device (Planmeca Oy, Helsinki, Finland) before surgery and 6 months following the augmentation procedure.

### Surgical procedure

#### Split-thickness flap preparation

**Fig. 1:**
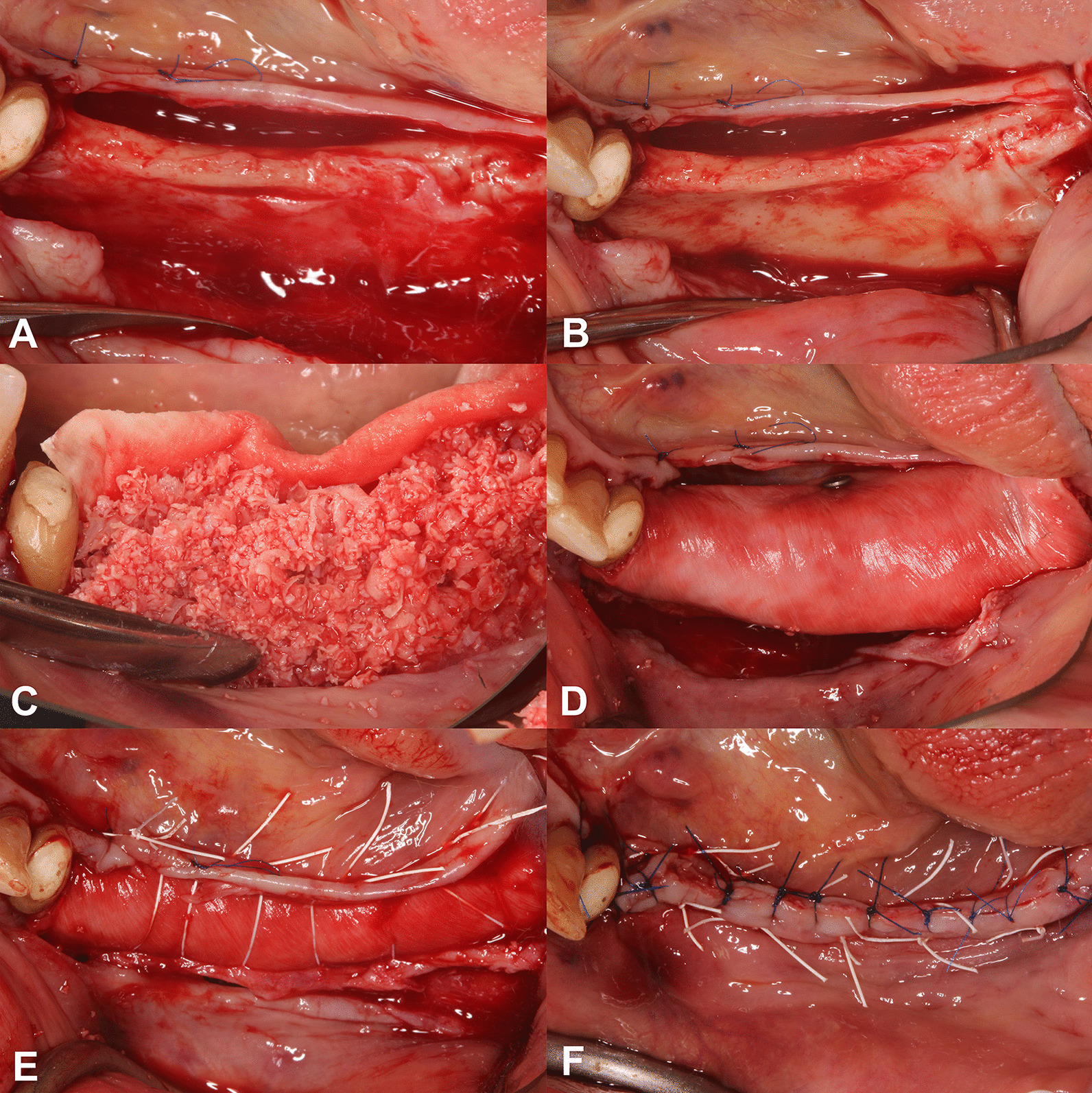
Demonstration of the surgical procedure. **A**: Mucosal layer preparation. **B**: Periosteal layer preparation. **C**: Application of the composite graft material. **D**: Membrane coverage. **E**: Periosteal sutures. **F**: Mucosal sutures

After local anesthesia, a midcrestal incision was made along the edentulous ridge and extended to the gingival sulcus of two neighboring teeth for better accessibility and to prevent unwanted lacerations. Vertical and periosteal releasing incisions were avoided to prevent periosteal blood supply disturbance. On the lingual aspect, full-thickness mucoperiosteal flap elevation was performed to the level of the mylohyoid line. Muscles and fibers were released from the inner surface of the flap, resulting in the buccal displacement of the lingual flap. On the buccal side, full-thickness preparation was carried out from the midcrestal incision to the mucogingival junction (MGJ), continued by partial thickness flap elevation with the use of surgical blades and tunneling instruments (Tunneling Knives®, Deppler, Rolle, Switzerland; Fig. 1a). After the separation of the mucosal layer, the periosteum was elevated from the bone surface by blunt dissection (Fig. 1b).

#### Graft preparation

Bone chips were harvested with a scraper (Safescraper Twist®, META Technologies S.R.L., Reggio Emilia, Italy) from the premolar, molar, and retromolar areas, without the preparation of a second surgical wound. Autogenous bone chips were collected in a sterile Petri dish, hydrated with sterile saline solution, and mixed with bovine-derived xenograft (BDX; Bio-Oss®, Geistlich, Wolhusen, Switzerland). A 50–50% composite graft of autogenous bone chips and BDX particles was applied to utilize the osteogenic properties of autogenous bone chips and the osteoconductive effect of BDX.

#### Membrane adaptation and bone grafting

A resorbable collagen membrane (Bio-Gide®, Geistlich, Wolhusen, Switzerland) was shaped according to the size of the surgical area. It was fixed on the lingual aspect with two or three titanium pins (Titan pin set, Botiss, Zossen, Germany) or membrane fixation screws (Pro-fix Precision Membrane Fixation System, Osteogenics Biomedical, Lubbock, Texas, USA). After the membrane was secured on the lingual side, the composite graft was compacted tightly against the residual alveolar crest (Fig. 1c). After grafting, the membrane was folded over the grafted area and secured on the buccal aspect with titanium pins (Fig. 1d).

#### Double-layer wound closure

The buccal periosteum was sutured with horizontal mattress sutures to the lingual full-thickness flap using a 3–0 non-resorbable expanded polytetrafluoroethylene suturing material (e-PTFE; Cytoplast sutures, Osteogenics Biomedical, Lubbock, Texas, USA; Fig. 1e). The buccal mucosal layer was then sutured to the lingual flap with horizontal mattress sutures and single interrupted sutures using a 4–0 non-resorbable e-PTFE suturing material (Cytoplast sutures, Osteogenics Biomedical, Lubbock, Texas, USA; Fig. 1f). The sutures were removed after 14 days.

#### Re-entry procedure and implant placement

**Fig. 2:**
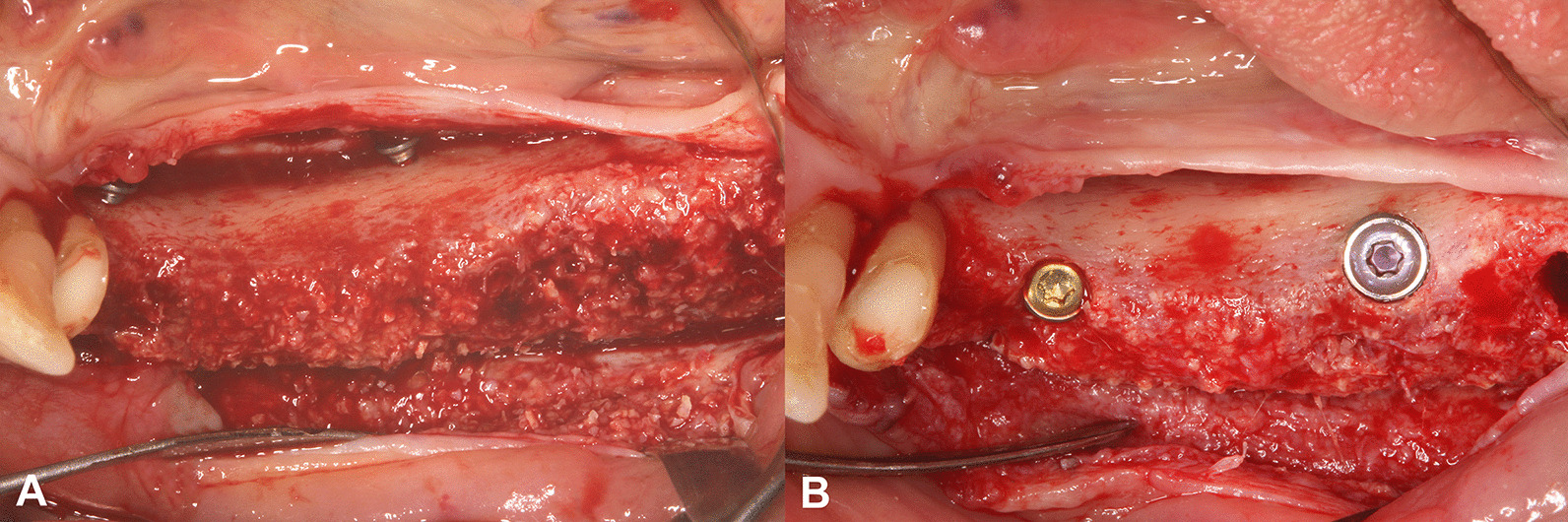
Clinical situation at re-entry surgery. **A**: 6-month follow-up situation. **B**: Positioned bone-level implants

After a 6-month healing period, guided implant placement was planned according to a follow-up CBCT scan. Subsequently, the re-entry procedure was performed, during which dental implants with a minimum diameter of 4 mm were inserted (Fig. 2).

### Three-dimensional radiographic analysis

#### Segmentation

**Fig. 3:**
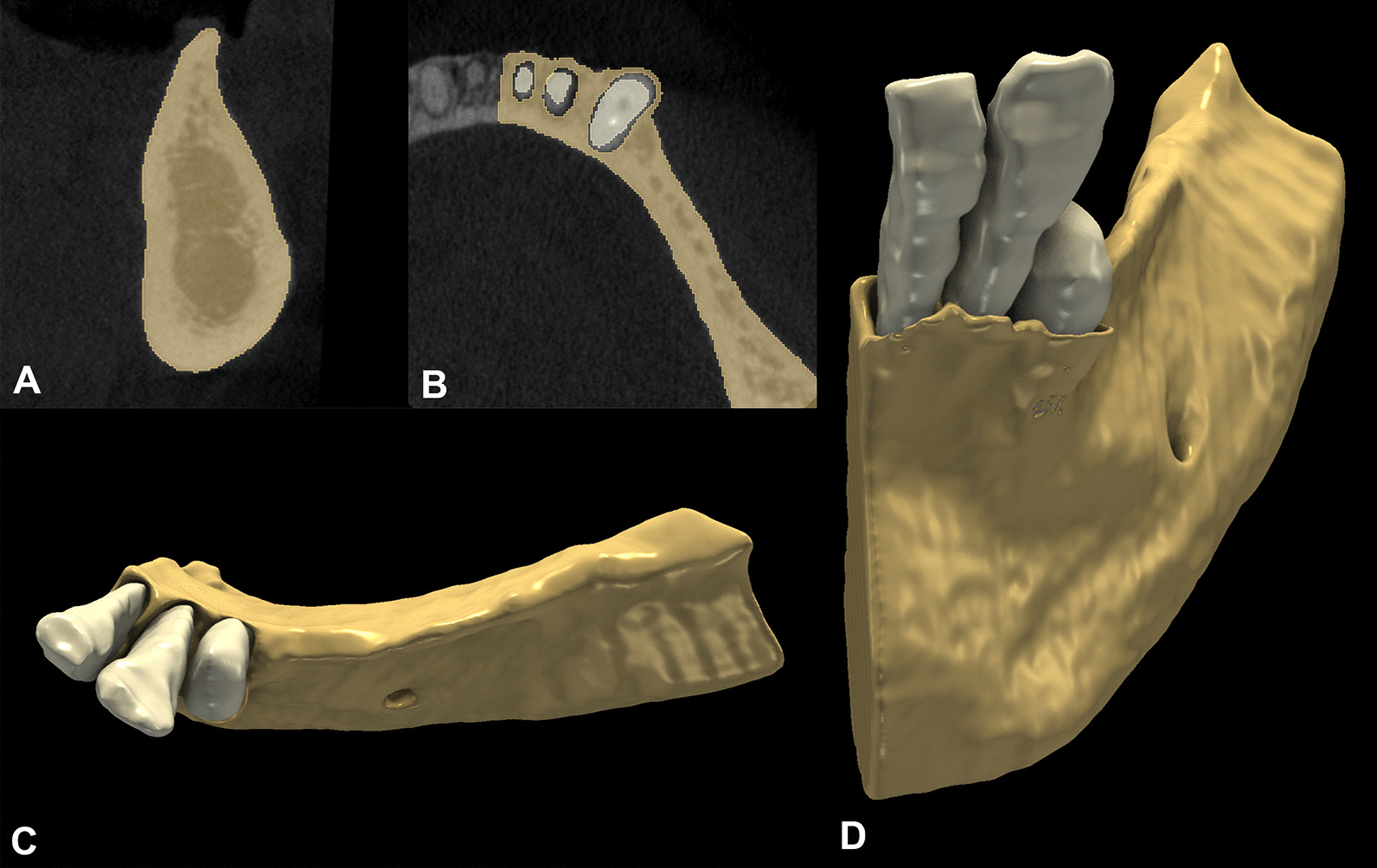
Baseline alveolar ridge morphology. **A**: Sagittal alveolar ridge outline. **B**: Axial alveolar ridge outline. **C**–**D**: 3D morphology of the alveolar ridge defect

**Fig. 4:**
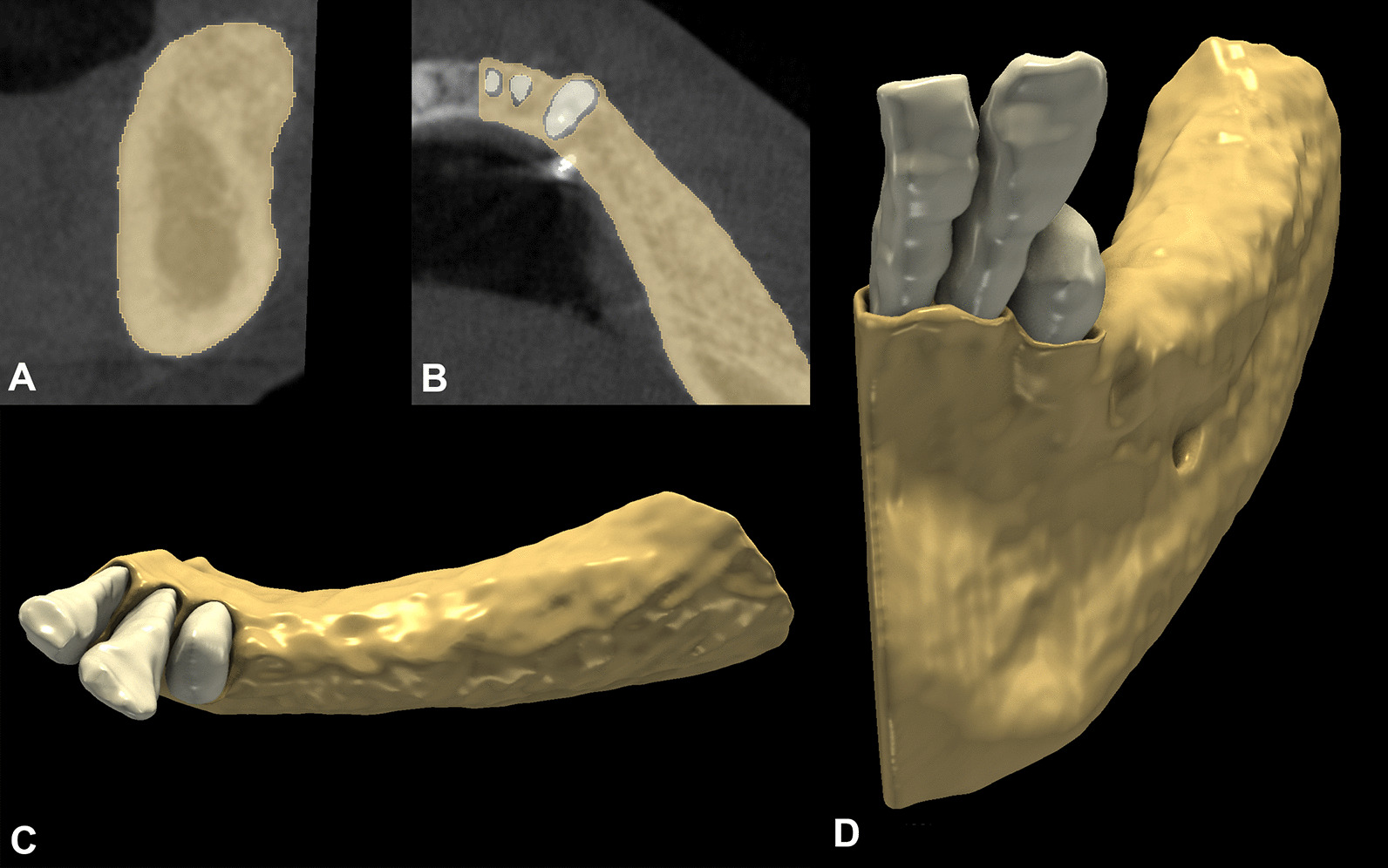
6-month follow-up alveolar ridge morphology. **A**: Sagittal alveolar ridge outline. **B**: Axial alveolar ridge outline. **C**–**D**: 3D morphology of the reconstructed alveolar ridge

Baseline and 6-month follow-up CBCT scans were imported into an open-source Digital Imaging and Communications in Medicine (DICOM) imaging software platform (3D Slicer). Using a dedicated semi-automatic image segmentation method, teeth and alveolar bone were individually segmented at the surgical area both pre- and postoperatively. Subsequently, the CBCT scans were reconstructed into 3D virtual models. The segmentation method comprised the following steps: (i) delineation of anatomic structures on every 10^th^ slice to generate initial binary labelmaps; (ii) morphological contour interpolation was performed to calculate interim missing labelmaps; (iii) smoothing and correction of minor errors with real-time 3D rendering. Segmentation was performed on both the baseline and the 6-month follow-up CBCT scans (Figs. 3 and 4.).

#### Volumetric hard tissue alterations: 3D subtraction analysis

**Fig. 5:**
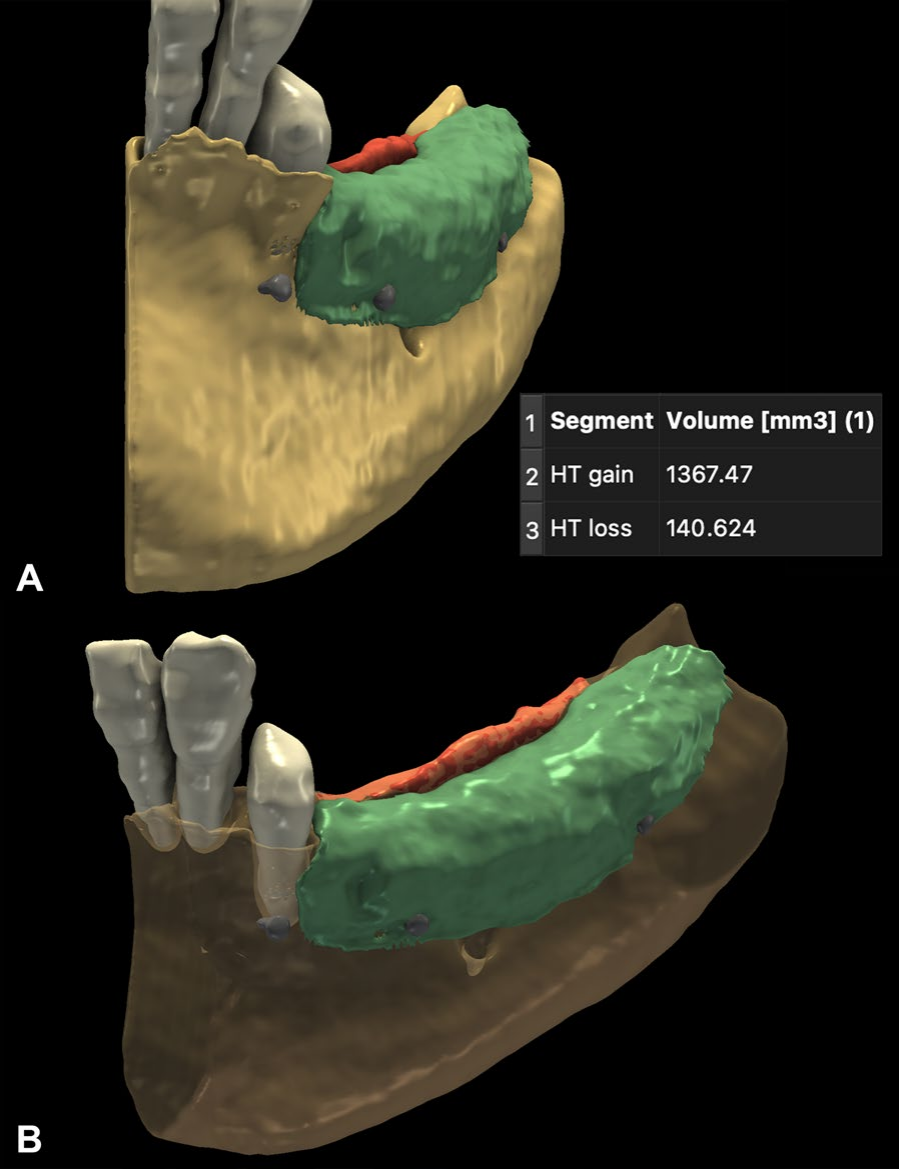
Evaluation and quantification of volumetric hard tissue changes. **A**–**B**: Hard tissue gain is visualized in green, and hard tissue loss is visualized in red

Following image segmentation, spatial registration of DICOM datasets was carried out automatically, using a voxel intensity-based registration method. The accuracy of the superimposition was controlled by changing the color palette of the CBCT scans from gray to red (baseline) and green (6-month follow-up). To visualize hard tissue gain, preoperative 3D models were subtracted from the postoperative 3D models. In contrast, to visualize occasional hard tissue resorption, postoperative 3D models were subtracted from the preoperative models (Fig. 5). Volumetric differences between pre- and postoperative CBCT scans were calculated in the Segment statistics module of 3D Slicer and expressed in cubic millimeters (mm^3^).

#### Validation of augmentation efficacy: volume-to-surface ratio calculation

**Fig. 6:**
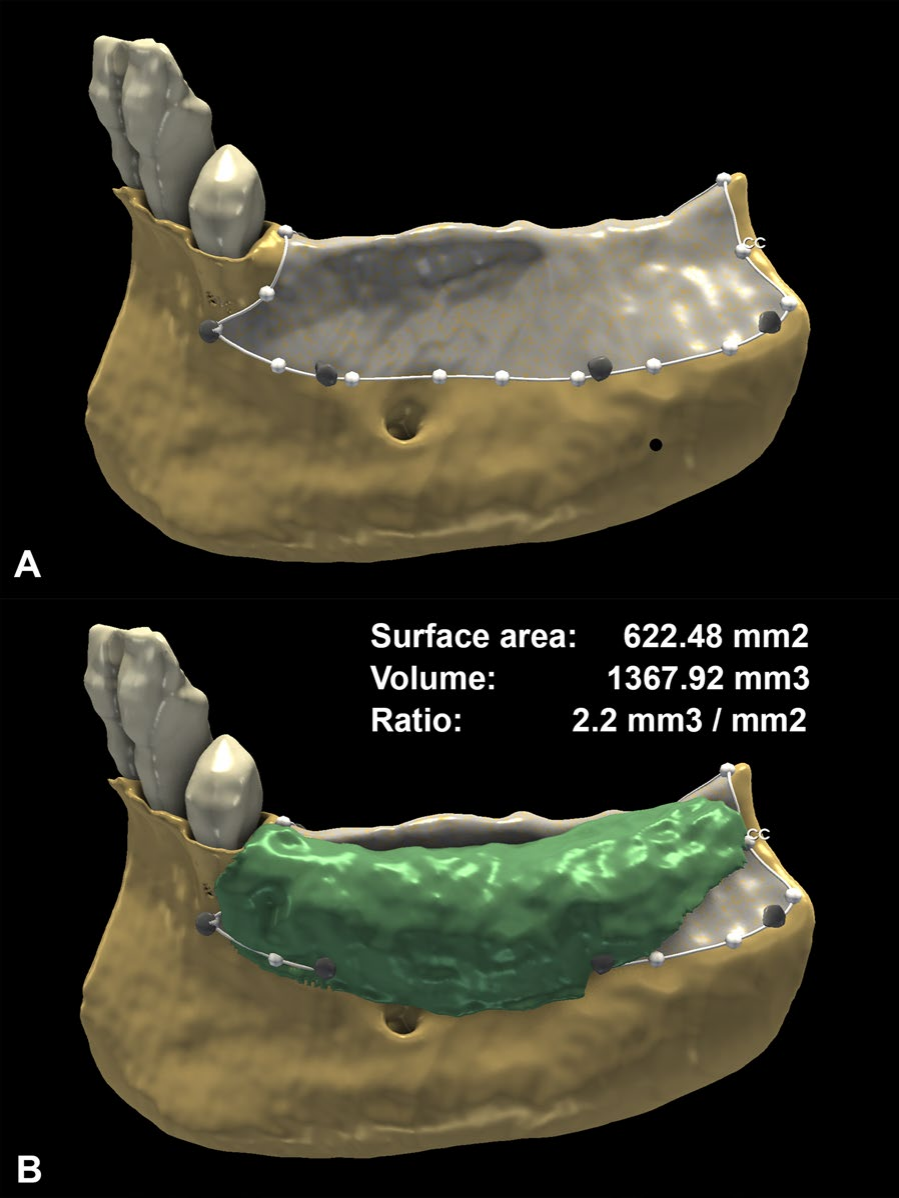
Efficacy of the augmentation. **A**: Surface area of the augmentation outlined by the titanium pins. **B**: Calculation and illustration of the volume-to-surface ratio

To determine the efficacy of the augmentation independently from the size of the surgical area, the volume-to-surface ratio was defined as a standardized measurement value. This ratio shows the average volumetric hard tissue gain per surface area. The bone surface area, where the augmentation was performed, was outlined by placing markup points at the titanium pins visible on the postoperative CBCT scans and tracing a closed curve markup. Using the curve cut function of the dynamic modeler module of 3D Slicer, a new surface model was generated from the preoperative alveolar bone model representing the augmented area (Fig. 6a). The bone surface area was expressed in square millimeters (mm^2^). The volume-to-surface ratio was calculated with the following formula (Fig. 6b): **volumetric hard tissue gain (mm**^**3**^**)/surface area of augmentation (mm**^**2**^**)**.

### Linear hard tissue changes—Planar grid method

**Fig. 7:**
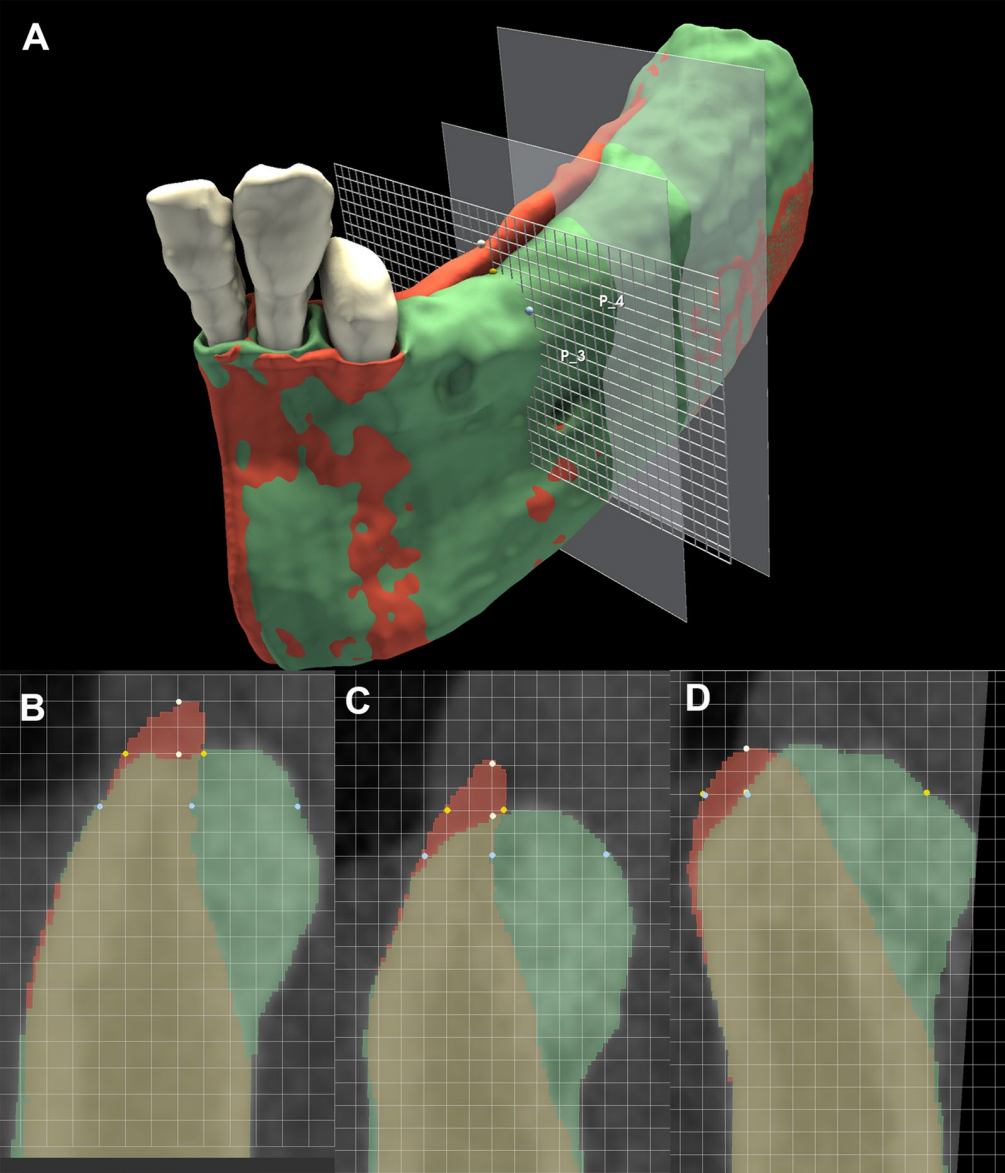
Linear measurements using a planar grid. **A**: 3D visualization of the measurement process. **B**–**D**: Linear measurements performed in three planes (yellow dot: baseline ridge width 2 mm apical to the baseline alveolar crest; blue dot: measurement of the horizontal ridge changes 2 mm apical to the follow-up alveolar crest; white dot: midcrestal vertical hard tissue loss)

CBCT images were reoriented so that the sagittal axis became parallel, and the coronal axis became perpendicular, to the alveolar ridge at the surgical area. Consequently, a cross-sectional view was acquired in the coronal view window where the linear measurements were performed. The linear hard tissue changes were measured in three planes at each surgical site (mesial: first plane; middle: second plane; distal: third plane). The mesial and distal endpoints of the augmentation were marked by the most mesially and distally placed titanium pins, then three planes were generated between the two endpoints at equal distances. On the coronal slice view, a grid with a 1-mm interval was overlaid on the image. The horizontal linear measurements were done at two levels. Firstly, the baseline alveolar ridge width was determined 2 mm apical from the marginal crest of the preoperative situation. Secondly, the follow-up alveolar ridge width was measured 2 mm apical from the marginal crest of the postoperative situation. Additionally, the horizontal linear hard tissue changes were measured at the second level. Midcrestal vertical measurements were performed to determine the value of occasional marginal bone resorption. An average value was calculated from measurements in all three planes for all investigated parameters (Fig. 7).

#### Analysis of morphological alterations: colormap analysis

**Fig. 8:**
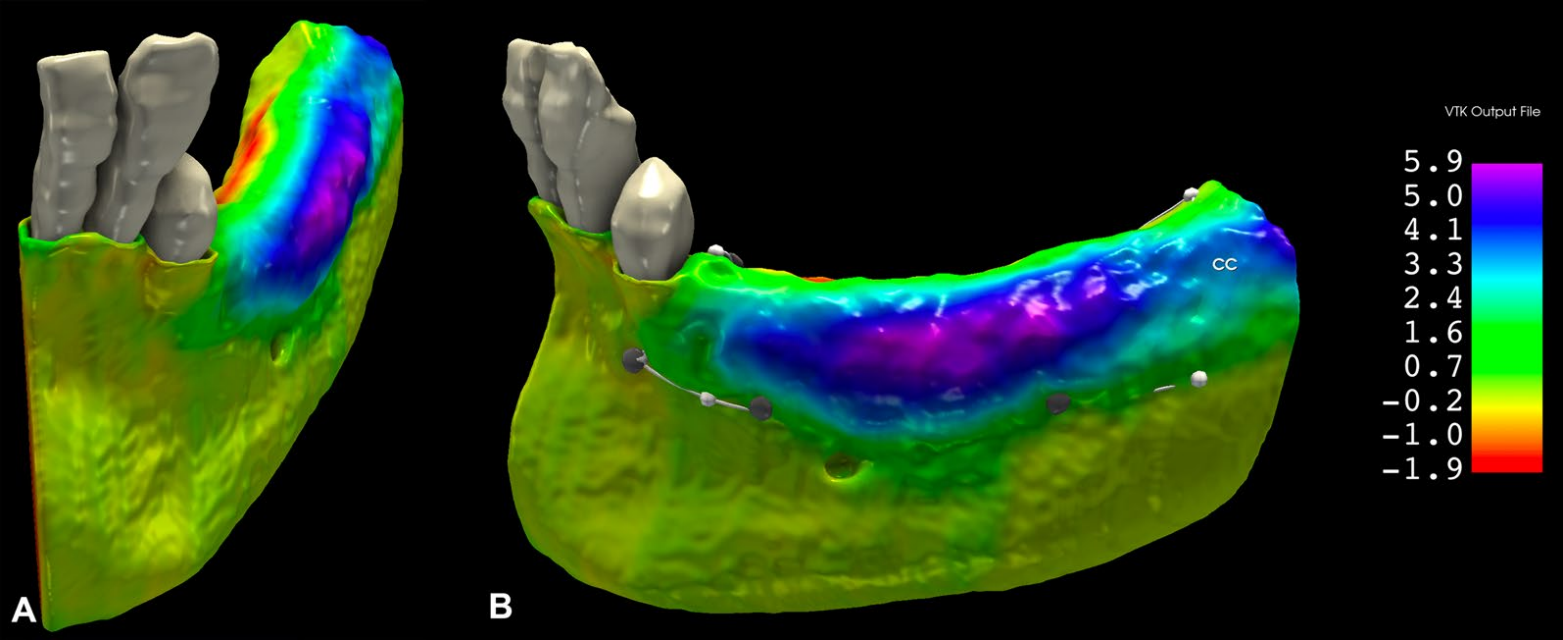
Assessment of morphological changes using a 3D colormap. **A**–**B**: Color-coded VTK file visualizes the closest distance between two points of the input models. The greatest positive distance (hard tissue gain) is visible in purple, and the greatest negative distance (hard tissue loss) is visible in red

For a visual demonstration, colormaps were used to subjectively analyze the areas with the greatest hard tissue gain and loss. In 3D Slicer, the Model to Model distance module [33] was used to generate a VTK file (Visualization Toolkit) showing the color code based on the shortest distance between points (signed shortest distance). The largest positive distance was visible in dark blue, the largest negative distance was visible in red, and the zero distance was visible in green (Fig. 8).

### Outcome variables

The primary outcome of the investigation was the volumetric hard tissue alteration assessed by subtraction analysis.

The secondary outcomes of the study were to (i) standardize the efficacy of horizontal GBR procedures using the volume-to-surface ratio, (ii) evaluate linear horizontal hard tissue changes in three planes of each augmented site using a planar grid measurement method, and (iii) analyze 3D morphological alterations using a colormap model to acquire an in-depth understanding of postoperative healing mechanics.

### Statistical analysis

Descriptive statistical analysis was performed for all the variables, and data were expressed as mean values ± standard deviation. Absolute baseline and follow-up hard tissue volumes were not determined, so only descriptive statistics were used for this variable. Due to the uneven distribution of the values and the limited sample size, non-parametric statistical analysis was performed for all the variables. Spearman’s rank order correlation was performed to determine the level of correlation between the surface area of the augmentation and the volume-to-surface ratio to investigate whether the surgical area size influenced the surgery results. Spearman’s rank order correlation was also carried out to investigate the influence of the participant’s age on the surgical outcomes. The level of significance between baseline and 6-month follow-up horizontal alveolar ridge width values was determined by the Wilcoxon matched-pairs test. All statistical calculations were performed in SPSS Statistics® (IBM, Armonk, New York, USA).

## Results

### Patient and site demographic data

In the present study, eight patients with 10 surgical sites were enrolled, with two patients presenting contralateral defects. All participants were female, with a mean age of 59.20 ± 11.16 years. Of the 10 treated sites, seven were located on the left, and three were located on the right lateral region.

### Volumetric hard tissue changes

With 3D subtraction analysis, an average of 605.32 ± 380.68 mm^3^ volumetric hard tissue gain was detected as a result of horizontal GBR. With thorough analysis, hard tissue resorption was also detected at the top of the edentulous ridge and the lingual aspect of the surgical area in all the evaluated cases. The amount of hard tissue resorption averaged 238.48 ± 127.82 mm^3^ (Table 1).

**Table 1:** Volumetric hard tissue gain and hard tissue loss

Surgical site	Hard tissue gain (mm^3^)	Hard tissue loss (mm^3^)
1	670.54	161.84
2	736.14	106.18
3	202.06	406.05
4	742.02	293.77
5	1367.47	140.62
6	223.36	81.25
7	150.17	118.21
8	767.74	353.69
9	331.21	352.22
10	862.50	370.95
Average ± st. deviation	605.32 ± 380.68	238.48 ± 127.82

### Efficacy assessment of horizontal ridge augmentation

Due to the varying size of the surgical area, the efficacy of the surgical procedure in each case was determined by the volume-to-surface ratio. The mean surface area of augmentations demarcated by titanium pins was 489.32 ± 186.44 mm^2^. The volume-to-surface ratio, calculated by the formula described in the materials and methods section, averaged 1.19 ± 0.52 mm^3^/mm^2^ (Table 2).

**Table 2:** Efficacy of augmentation determined by the volume-to-surface ratio

Surgical site	Volumetric hard tissue gain (mm^3^)	Surface area (mm^2^)	Volume-to-surface ratio (mm^3^/mm^2^)
1	670.54	402.81	1.66
2	736.14	540.23	1.36
3	202.06	398.27	0.51
4	742.02	691.53	1.07
5	1367.47	622.48	2.2
6	223.36	174.54	1.28
7	150.17	289.11	0.52
8	767.74	764.38	1.00
9	331.21	403.55	0.87
10	862.50	606.29	1.42
Average ± st. deviation	605.32 ± 380.68	489.32 ± 186.44	1.19 ± 0.52
*p*-value*	0.405 (r_s_ = 0.29**)		

*Significance level of correlation between surface area and volume-to-surface ratio

**Spearman’s correlation coefficient

Based on the non-parametric Spearman’s rank order correlation, a weak positive correlation existed between the size of the surgical area and the volume-to-surface ratio (correlation coefficient: r_s_ = 0.29), but it was non-significant (p > 0.05).

The correlation between age and the efficacy of the augmentation, indicated by the volume-to-surface ratio, was negligible (correlation coefficient: r_s_ = − 0.1) and non-significant (p > 0.05).

### Linear hard tissue changes

The baseline alveolar ridge width, measured 2 mm apically from the marginal crest of the preoperative situation, averaged 4.38 ± 1.04 mm. The follow-up alveolar ridge width 2 mm apically from the alveolar crest of the postoperative situation averaged 7.33 ± 0.81 mm. A statistically significant difference existed between the baseline and follow-up horizontal ridge width values (p = 0.005; Table 3). Data on all the recorded linear measurements are included in Additional file 1: Tables S1, S2, S3 and S4.

**Table 3:** Baseline and follow-up horizontal ridge dimensions

Surgical site	Baseline crestal ridge width (mm)	Baseline ridge width at the level of follow-up measurements (mm)	Follow-up ridge width (mm)
1	4.83	5.33	9.17
2	4.50	4.50	7.5
3	5.00	5.67	6.67
4	4.67	5.67	8.00
5	3.33	3.5	7.83
6	5.83	5.83	7.00
7	5.67	6.17	6.67
8	2.67	2.67	7.00
9	4	6.33	6.83
10	3.33	3.83	6.67
Average ± st. deviation	4.38 ± 1.04	4.95 ± 1.25	7.33 ± 0.81
*p*-value*	0.005		

*significance level between pre- and postoperative linear measurements

Presented values are calculated as an average of linear measurements performed on three planes at each surgical site

The postoperative measurements were taken slightly more apical to the preoperative measurements due to the crestal hard tissue resorption. Horizontal linear hard tissue changes were measured at this second, more apical level. The horizontal hard tissue gain averaged 3.00 mm ± 1.45 mm, whereas the average horizontal hard tissue resorption was 0.72 mm ± 0.48 mm. The midcrestal vertical hard tissue loss averaged 1.18 mm ± 0.81 mm (Table 4).

**Table 4:** Linear alveolar ridge alterations following horizontal GBR

Surgical site	Horizontal hard tissue gain (mm)	Horizontal hard tissue loss (mm)	Crestal vertical hard tissue loss (mm)
1	5.00	1.17	1.00
2	2.83	0.17	0.00
3	2.17	1.50	2.17
4	2.83	0.50	1.17
5	4.83	0,50	1.83
6	1.83	1.00	0.17
7	0.83	0.33	0.33
8	4.33	0.00	1.17
9	1.50	1.00	2.00
10	3.83	1.00	2.00
Average ± st. deviation	3.00 ± 1.45	0.72 ± 0.48	1.18 ± 0.81

Presented values are calculated as an average of linear measurements performed on three planes at each surgical site

### Morphological alterations

On the 3D colormap analysis, a slight lingual and crestal hard tissue resorption was observed in all of the 10 cases to a varying extent. In certain instances, the greatest extent of hard tissue gain was observed 2–3 mm apical to the initial level of the marginal crest.

## Discussion

Radiographic evaluation of the 10 healed edentulous sites treated with horizontal GBR using a split-thickness flap design, composite graft materials, and a resorbable collagen membrane was carried out with a novel CBCT-based subtraction analysis. The main focuses of this examination were to validate the efficacy of the novel surgical approach and describe a 3D radiographic evaluation method by evaluating hard tissue alterations in cases that were part of a larger ongoing randomized controlled clinical trial.

Previously, our group evaluated volumetric and 3D morphological changes of hard tissue alterations after alveolar ridge preservation and regenerative periodontal therapy [29, 30]. Similarly, in this study, volumetric evaluation was performed by subtracting pre- and postoperative 3D models [34]. Although volumetric hard tissue changes could be determined, due to the different augmentation area sizes, volumetric values varied between cases. To overcome this limitation, the area of augmentation, marked by the positions of the titanium pins, was calculated, and the efficacy of the procedure was determined by the volume-to-surface ratio. This method allowed us to validate the effectiveness of the surgical treatment, independent of the surgical area sizes. Besides 3D quantitative analysis, 3D morphological alterations were assessed. Although expressing these types of changes numerically is much more difficult, it provides clinically more relevant information on tissue healing.

As the result of the statistical analysis indicated, no significant correlation existed between the augmentation area and the volume-to-surface ratio. This means that the procedure was equally effective in all cases regardless of the surgical area size.

Most frequently, horizontal hard tissue changes are validated with radiographic or direct clinical linear measurements performed 2 mm apical to the alveolar crest at implantation sites. Thus, the overall horizontal change is calculated mathematically, but exact information on horizontal hard tissue gain and eventual hard tissue loss cannot be assessed. In the current study, spatial registration allowed us to assess the pre- and postoperative CBCT scans simultaneously on each image of the datasets. Binary labelmaps outline the pre- and postoperative alveolar ridge morphology on planar CBCT images, allowing for easier analysis. Due to the hard tissue resorption, the baseline horizontal measurements had to be performed at two levels: (i) 2 mm apical to the initial alveolar crest to determine the baseline ridge width and (ii) 2 mm apical to the follow-up alveolar crest to determine horizontal hard tissue gain. In this second level, the baseline width of the alveolar ridge was usually wider, and values did not accurately represent the initial situation.

The results in this investigation using a split-thickness flap design are comparable to data reported on horizontal GBR using a full-thickness flap design [11]. In two studies investigating horizontal hard tissue gain in the treatment of knife-edge ridges [13, 35], the authors reported greater horizontal hard tissue gain than in this current investigation, whereas the follow-up alveolar ridge width values were similar at 7.87 mm in Urban et al. [13] and 8.09 mm in Meloni et al. [35].

With the applied 3D evaluation method, slight hard tissue resorption at the lingual and midcrestal aspects of the surgical area could be demonstrated. In different animal studies, authors have demonstrated that the elevation of either full- or split-thickness flaps induces osteoclast activity that causes subsequent bone resorption [36–41]. Similar vertical and horizontal bone loss was observed by Saleh et al. [42] after full-thickness flap elevation during implant placement. In the case of horizontal GBR, similar bone resorption has not previously been described. Notably, bone resorption would have occurred at the buccal aspect as well, but the buccal grafting masked it on the radiographic images. In certain instances, the greatest extent of horizontal hard tissue gain was observed 2–3 mm apical to the baseline marginal crest. This leads to the assumption that the graft either (i) was apically displaced during surgery (e.g., when folding the membrane from the lingual to the buccal aspect) or (ii) shifted apically during the healing period. The alveolar bone crest showed a resorption tendency down to the most coronal point of the grafted area.

## Conclusions

With the application of this 3D analysis, horizontal alveolar ridge augmentation procedures using the GBR approach could be evaluated thoroughly, and previously unreported events occurring after healing could be demonstrated. This study showed the occurrence of lingual and midcrestal bone resorption, most likely caused by increased osteoclast activity after the elevation of the periosteum and undesired apical migration of the graft material. Additionally, with the application of the volume-to-surface ratio, the efficacy of the augmentation procedure could be evaluated independently of the surgical area size. The radiographic results achieved with a split-thickness flap design were comparable to those found in the literature. The radiographic changes occurring after horizontal GBR were examined and described on a limited sample size. Although statistical analysis was performed to draw further conclusions on the radiographic hard tissue changes, the entire sample of the ongoing prospective study must be evaluated.

## Supplementary Information

**Additional file 1.** Linear measurements.

## Data Availability

All data generated or analyzed during this study are included in this published article.

## Abbreviations

ARP: Alveolar ridge preservation
GBR: Guided bone regeneration
CBCT: Cone-beam computed tomography
FMBS: Full mouth bleeding score
FMPS: Full mouth plaque score
MGJ: Mucogingival junction
BDX: Bovine-derived xenograft
e-PTFE: Expanded polytetrafluoroethylene
DICOM: Digital Imaging and Communications in Medicine
VTK: Visualization Toolkit
